# Actinomycosis of Distal Phalanx Twenty Years after Flap Reconstruction of Index Finger: A Case Report

**DOI:** 10.5704/MOJ.1803.011

**Published:** 2018-03

**Authors:** N Prashant, A Azuhairy

**Affiliations:** Orthopaedic Oncology Unit, Hospital Pulau Pinang, Georgetown, Malaysia

**Keywords:** actinomycosis, flap surgery, chronic hand infections, osteomyelitis phalanx

## Abstract

Actinomycosis is a chronic granulomatous suppurative infection caused by anaerobic bacteria from genus Actinomyces which are normal flora of mouth, colon and vagina. Actinomycosis of upper extremity is rare. We report a case of actinomycosis of the distal phalanx of finger many years after flap reconstruction. The patient presented with two months’ history of chronic discharging sinus from the tip of his right index finger, which had sustained a degloving injury 20 years previously. It had been treated with an anterior chest wall flap which had healed uneventfully but was bulky due to excess tissue from the donor site. Radiograph revealed osetomyelitis changes of distal phalanx. Debulking surgery with curettage of the distal phalanx was done. Wound healing was uneventful. He was treated with six weeks of metronidazole and ciprofloxacin. The discharge from the distal phalanx cultured *actinomycosis odontolyticus*. Histopathology of the debrided tissue showed chronic inflammation. As far as we are aware, there are no reports of actinomycosis in a flap involving the finger treated previously with a chest wall skin flap. The infection was probably dormant for many years before manifesting as a discharging sinus. Although the finger flap was bulky, it was not problematic until it started to have serous discharge. With a thorough debridement of all infected tissue, six weeks of antibiotic was adequate. Ciprofloxacin was prescribed based on discharge culture sensitivity. Metronidazole was added as actinomycosis is anaerobic. Response was prompt as patient was not immunocompromised. At follow-up six months post-surgery the finger had recovered with good function. If not for the discharging sinus, patient would probably have tolerated his bulky finger for the rest of his life.

## Introduction

**Fig. 1:**
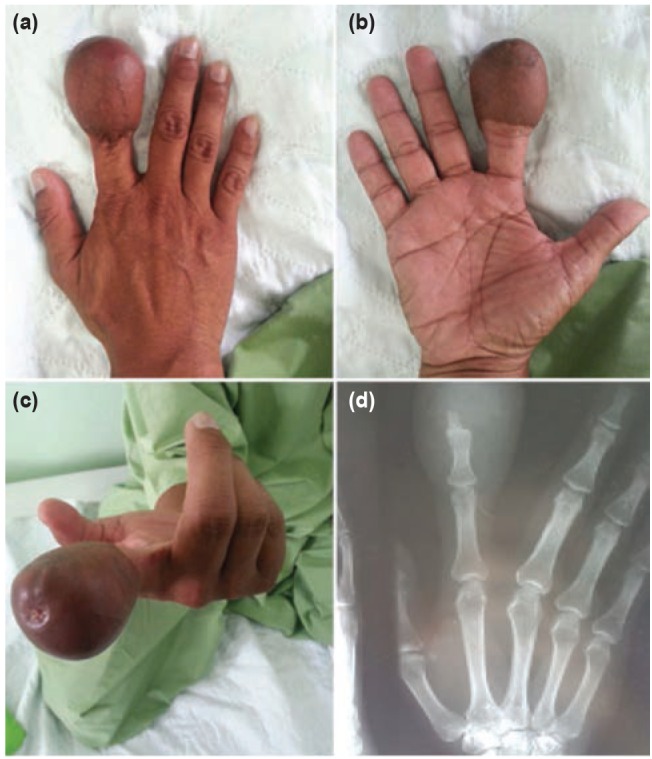
Preoperative images of (a) Dorsal, (b) Volar, (c) Fingertip and (d) Radiograph of the index finger with bulky soft tissue from previous skin flap.

Actinomycosis is a chronic granulomatous suppurative infection caused by anaerobic bacteria from genus Actinomyces which is a normal flora of mouth, colon and vagina^1^. Actinomycosis of the extremity is rare, even more so in the upper extremity^2^. We report an interesting case of actinomycosis of distal phalanx of finger many years after flap reconstruction (Fig. 1a, b).

## Case Report

Our patient presented with a chronic discharging sinus from the tip of his right index finger (Fig. 1c). Interestingly, this finger had sustained a degloving injury 20 years earlier and had been treated with an anterior chest wall flap. After two weeks, the flap was detached from the chest wall and the wound over the right index finger healed uneventfully; however, the finger was bulky due to excess tissue from the donor site. A month prior to presentation, the index finger started discharging serous fluid from the tip. There was no significant history of trauma at this time which preceeded the sinus formation. Radiograph of hand revealed osetomyelitis changes over the distal phalanx of the index finger (Fig. 1d). ESR was 78 mm/hr, suggestive of chronic inflammation.

**Fig. 2:**
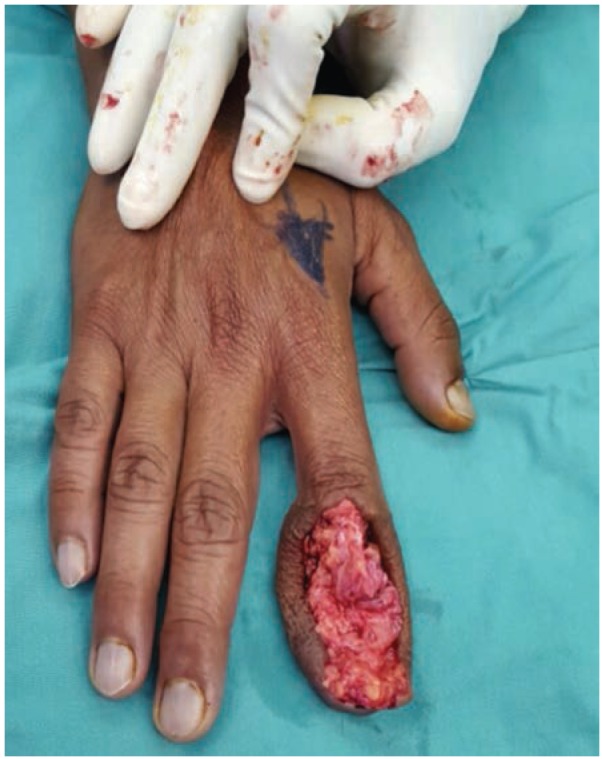
Intraoperative findings of dense fibrous tissue surrounding the osteomyelitic phalanx.

**Fig. 3:**
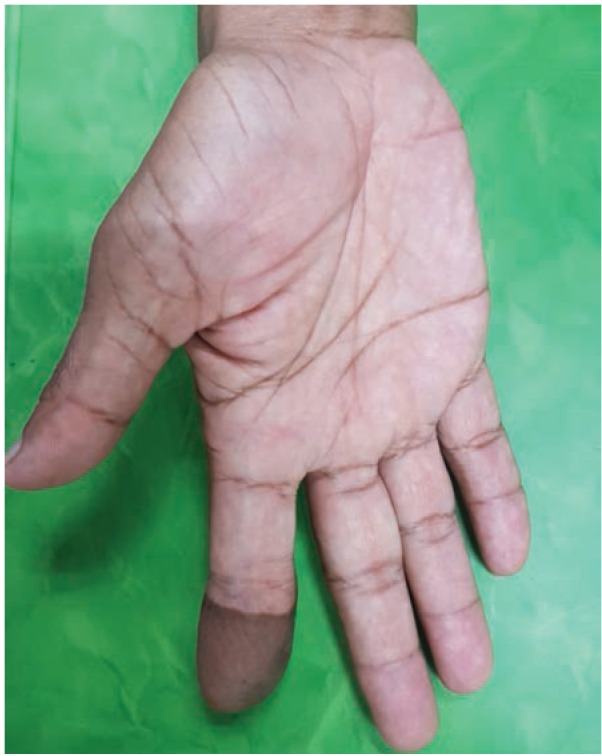
Volar view of index finger a year after surgery showing significantly less bulky soft tissue as a result of debulking.

He underwent a debulking surgery with curettage of the distal phalanx (Fig. 2). Intraoperatively, we noted the osteomyelitic changes in the bone surrounded by dense inflamed tissue which was further surrounded by relatively normal looking fatty tissue, probably form the donor flap. The fingertip was debulked to give it a more acceptable appearance. The wound healed within two weeks as expected and he was treated with six weeks of oral antibiotics. *Actinomycosis odontolyticu* was cultured from the tissue from the distal phalanx. The histopathology of the debrided tissue showed non-specific chronic inflammatory process. At six months, the index finger had completely healed with no sign of residual infection and with improved function compared to before surgery (Fig. 3).

## Discussion

Infection due to actinomycosis is rare and involvement of the upper limb is even more rare^2^. As far as we are aware, there is no report of actinomycosis in a previous skin flap reconstruction involving the finger. We believe the infection has been indolent for some time and manifested as a sinus many years later. As our patient was using his finger for most daily activities with very little limitation of function, we believe he would have contracted the infection during his daily use of the finger. Although the finger was bulky, our patient did not find it to be a problem until it started to have serous discharge from the tip of the finger. As the radiograph suggested osteomyelitis changes over the tip of distal phalanx, debridement and curettage of the bone became necessary and the opportunity was taken to debulk the excess tissue covering the index finger at the same time. Most literature on the infection propose antibiotics for a prolonged period of three months to a year^1,2^; however we instituted the antibiotic therapy for six weeks only. We believed with a through debridement of all infected tissue the six weeks duration was adequate. The choice of antibiotic was ciprofloxacin based on culture sensitivity result. Metronidazole was added as actinomycosis is an anaerobic bacterial infection. Response was prompt as our patient was healthy and most importantly not immunocompromised. We believed the longer antibiotic therapy of at least three months was probably appropriate for immunocompromised patients and in cases where the debridement was not done or there was a possibility of residual infection post-debridement.

On our follow-up a year post-surgery the finger wound had healed completely with improvement in the function compared to before debridement, with almost full range of motion. If not for the actinomycosis infection presenting with the discharging sinus, this patient probably would have just tolerated his bulky finger for the rest of his life.

## Conflict of Interest

The authors declare no conflicts of interest.
